# Moss occurrences in Yugyd Va National Park, Subpolar and Northern Urals, European North-East Russia

**DOI:** 10.3897/BDJ.7.e32307

**Published:** 2019-04-01

**Authors:** Galina Zheleznova, Tatyana Shubina, Svetlana Degteva, Ivan Chadin, Mikhail Rubtsov

**Affiliations:** 1 Institute of Biology of Komi Scientific Centre of the Ural Branch of the Russian Academy of Sciences, Syktyvkar, Russia Institute of Biology of Komi Scientific Centre of the Ural Branch of the Russian Academy of Sciences Syktyvkar Russia

**Keywords:** dataset, moss occurrences, Bryophyta, field study, herbarium data, literature reports, data paper

## Abstract

**Background:**

This study produced a dataset containing information on moss occurrences in the territory of Yugyd Va National Park, located in the Subpolar and Northern Urals, European North-East Russia. The dataset summarises occurrences noted by long-term bryological explorations in remote areas of the Subpolar and Northern Urals from 1943 to 2015 and from studies published since 1915.

The dataset consists of 4,120 occurrence records. The occurrence data were extracted from herbarium specimen labels (3,833 records) and data from published literature (287 records). Most of the records (4,104) are georeferenced.

A total of 302 moss taxa belonging to 112 genera and 36 families are reported herein to occur in Yugyd Va National Park. The diversity of bryophytes in this National Park has not yet been fully explored and further exploration will lead to more taxa.

**New information:**

A total of 4,120 moss occurrences records in the territory of Yugyd Va National Park were published.

## Introduction

Yugyd Va National Park is the largest protected area in the Komi Republic. The area of Yugyd Va is 18,941 km^2^ or 35% of the total area of all the Komi Republic’s protected areas. Yugyd Va National Park and Pechora-Ilych Nature Reserve (the latter being adjacent to the southern part of the National Park) were recognised by UNESCO as a World Heritage Site, the Virgin Komi Forests, in 1995. This territory is a remnant of the largest intact forest area in Europe. This area is a refugium for many animal and plant species that are endangered or have disappeared in other places and functions as a source of genetic diversity for many boreal species’ populations (Degteva and Ponomarev 2014).

The earliest data on the mosses of Yugyd Va National Park were published by the famous Russian botanist R.R. Pole (Pole 1915). His investigations were carried out in 1905 and 1907 in the Shchugor River basin in the Northern Urals. Later, Y. Tsinserling in 1926 and P.L. Gorchakovskii in 1954 collected mosses in the vicinity of Mount Sablya in the Subpolar Urals (Tsinzerling 1935, Gorchakovskiy 1958). V.B. Kuvaev collected mosses on the mountain slopes in the basins of the Shchugor River in the Northern Urals and the Kozhim River in the Subpolar Urals in 1948-1949 (Kuvaev 1970). A.P. Dyachenko conducted botanical research in 1984 on Mount Narodnaya in the Kosyu River basin in the Subpolar Urals (Dyachenko 1997, Dyachenko and Fomicheva 1986). I.D. Kildyushevskiy explored the vegetation cover of the Subpolar Urals in the Kozhym River basin in 1948-1951. Collections made in the course of these studies created the background for further accounts devoted to the moss flora of the Subpolar Urals (Kildyushevskiy 1956).

Specialists from the Institute of Biology of Komi Scientific Centre of the Ural Branch of the Russian Academy of Sciences began botanical exploration of Yugyd Va National Park in 1943. As a result, 3,829 moss occurrences were recorded from its territory by 2015 (or 93% of the known moss occurrences in the park). More than 99% of the moss samples from this protected area, stored in the SYKO Herbarium, were collected by specialists of this Institute. About 27% of the occurrences documented in the SYKO Herbarium were made by the authors of this article (Degteva S., Zheleznova G.). The following botanists took part in the identification of the mosses of Yugyd Va National Park stored in the SYKO Herbarium: Abramova A.L., Afonina O.M., Efimova S.F., Fedosov V.E., Ignatova E.A., Ignatov M.S., Kildyushevskiy I.D., Savich L., Shennikova M., Shlyakov R.N., Shubina T.P. and Zheleznova G.V. Authors of this paper (Zheleznova G.V., Shubina T.P.) had identified about 80% of the collection. The moss flora analyses of the Yugyd Va National Park were published in a series of works by Shubina 2007, Zheleznova 2010, Zheleznova and Shubina 2012, Zheleznova et al. 2015b, Zheleznova et al. 2015a, Zheleznova et al. 2016, Degteva 2016, Zheleznova and Shubina 2018, Zheleznova et al. 2013. Published datasets on moss occurrences in Russia, i.e. Database of Moss Flora of Russia (Ivanov et al. 2017), Cryptogamic Russian Information System (CRIS) (Melechin et al. 2013) and GBIF (GBIF.org 2018), do not contain information about moss records in the territory of Yugyd Va National Park.

Prepared according the concept of "data paper" (Chavan and Penev 2011, Penev et al. 2017), this paper aims to describe a dataset on moss occurrences in Yugyd Va National Park (Subpolar and Northern Urals) recently published by us in GBIF as a Darwin Core Archive (Zheleznova et al. 2018; see also Suppl. material 1).

## Project description

### Title

Moss occurrences in Yugyd Va National Park, Subpolar and Northern Urals, European North-East Russia

### Personnel

Svetlana Degteva, Tatyana Shubina

### Study area description

Yugyd Va National Park is the largest National Park in Europe and contains an array of primary boreal (Northern) forests preserved in an almost undisturbed state. The park is located on the western slopes of the Subpolar and the Northern Urals, on the border between Europe and Asia. The Urals are a mountain range that runs almost continuously along the 60°E meridian from the Arctic Ocean coast to the Ural River and north-western Kazakhstan. The Urals are a large and important biogeographic boundary that separates the flora and fauna of the continents of Europe and Asia (Chibilev 2011).

Most landscapes within these areas are free from anthropogenic impacts, so plant communities and their dynamics exist there in their natural state. Natural succession in these forests is initiated by such disturbances as windfalls and fires. Recreational uses of the park include rafting, boating and hiking in the summer and Nordic skiing in the winter. Due to the remote location of the park, the amount of tourism there is still quite low.

### Design description

The study of the flora and vegetation diversity of terrestrial ecosystems in the park was performed with an integrated approach at both community and species levels. The standard methods of plant systematics, geobotany and phytogeography were used. Important “Plant Areas” were studied by describing plant communities along altitudinal gradients from watercourses to their watersheds. The results of this study were documented in the Herbarium (SYKO) of the Institute of Biology of Komi Scientific Centre of the Ural Branch of the Russian Academy of Sciences.

A dataset was prepared from data published in scientific literature and acquired from the moss collection of the Herbarium (SYKO). The earliest collections examined came from 1943 and the most recent from 2015. For each species, information is given on the catalogue number, including herbarium label data from the moss collection of the Herbarium (SYKO), associated references, basis of record, geographic locality, verbatim locality, geographic coordinates, collection date, collector and person who identified the specimen (“identified by”).

### Funding

The Ministry of Education and Science of the Russian Federation. Project N 18-4-4-14 (АААА-А17-117112270073-0) of the Ural Branch of the Russian Academy of Sciences Complex Programme "Diversity of the main components of ecosystems along the latitudinal and altitudinal gradients of the Northern and the Subpolar Urals’ western macroslopes" and Project N АААА-А19-119011790022-1 "The diversity of flora and vegetation of the Subpolar Urals’ Western macroslope".

## Sampling methods

### Study extent

This study was carried out in the foothills and mountain landscapes of the Subpolar Urals, in the basins of four first-order tributaries of the Pechora River: the Kozhim River (in the Rosomakha, Maldynyrd, Zapadnye Saledy, Yuasnyrd, Maldyiz and Obeiz mountain ranges), the Kosyu River (in the Vostochnye Saledy and Kolokolenny mountain ranges), the Bolshaya Synya River (on Sablinskiy ridge) and the upper stream of the Shchugor River. The major tributaries of the Kozhim River studied were the Balbanyu River, Limbekoyu River and Syvyu River. In the Kosyu River basin, the territories near its major tributary (the Vangyr River) and near the lakes Mezhgornye and Okunevye were studied. In the basin of the upper stream of the Bolshaya Synya River, the areas adjacent to its tributaries, including the Voyvozh-Synya River and the Lunvozh-Synya River, were studied.

Mosses of the Northern Urals were collected in the basin of the Shchugor River (in the Telpos and Sumk-Ner mountain ranges and on the mountains Telpos-iz, Yank-Kart-Tump, Vay-Khury-Tump and Khodymalya-Tump) and two of its tributaries: the Podcherem River (Pelener Mountain) and the Telpos River.

### Sampling description

The authors contributing to the dataset used standard methods of mosses collection. The collecting localities were arbitrarily chosen in an attempt to include the largest number of different floristic associations and landscape forms in the samples. To achieve this purpose, a net of radial routes around each field base camp was planned. Short descriptions of plant communities were made in the localities of mosses collection. Moss samples were collected once and on each type of substrate.

In addition to the authors’ collections, the dataset includes information on moss occurrences obtained from literature (Dyachenko 1997, Dyachenko and Fomicheva 1986, Gorchakovskiy 1958, Kildyushevskiy 1956, Kuvaev 1970, Pole 1915, Tsinzerling 1935). More than 92% of the occurrence records were based on preserved samples from the Herbarium (SYKO) (Table 1). Some records (412) from locations adjacent to the border of the National Park were also included in the dataset.

### Quality control

The data were collected and identified by bryologists from the Institute of Biology of Komi Scientific Centre of the Ural Branch of the Russian Academy of Sciences. Some moss specimens were identified by taxonomic specialists from the Komarov Botanical Institute of the Russian Academy of Sciences, the Faculty of Biology of Lomonosov Moscow State University and the Tsitsin Main Botanical Garden of the Russian Academy of Sciences.

### Step description

On each herbarium label, the following fields were filled out: “Scientific name”, “Locality” (with geographic coordinates), “Habitat”, “Substrate”, “Collector name”, “Determined by” (identification), “Collection date” and “Catalogue Number”. For the data obtained from literature, the descriptions given by the author(s) were converted into these herbarium label fields when possible. The dataset fields’ names were chosen according to Darwin Core (Wieczorek et al. 2012) and include the following: «occurrenceID», «institutionID», «collectionCode», «catalogNumber», «associatedReferences», «basisOfRecord», «kingdom», «phylum», «class», «family», «genus», «scientificName», «specificEpithet», «scientificNameAuthorship», «infraspecificEpithet», «taxonRank», «country», «countryCode», «recordedBy», «day», «month», «year», «locality», «identifiedBy», «decimalLatitude», «decimalLongitude», «coordinatePrecision», «coordinateUncertaintyInMeters», «georeferencedBy», «geodeticDatum».

References to the published literature, from which data were obtained for the checklist compilation, are presented in the “citations” section of the metadata. The herbarium label data were taken from the moss collection of the Herbarium (SYKO). All occurrence records were merged into one Microsoft Excel worksheet. The species names given were determined according to the Check-list of mosses of East Europe and North Asia (Ignatov et al. 2006). The unique values from the “Species” field were used as a preliminary Yugyd Va National Park mosses checklist. The preliminary checklist was verified on the “taxonomic name resolution service” (Boyle et al. 2013) with the help of the “taxize” package in the R environment (Chamberlain and Szocs 2013).

In most cases, georeferencing was performed using paper maps of different scales. The maps were in the Kavrayskiy projection and SK-42 reference system. Maps with a 1:500000 scale were used for obtaining coordinates with 1-minute precision (3,903 occurrences). Maps with a scale of 1:100000 were used for obtaining 1-second-precision coordinates (269 occurrences). The remaining 22 occurrences were left ungeoreferenced because of the ambiguity of the locality description. All coordinates were transformed in the WGS 84 reference system with QGIS software. The coordinate uncertainty in metres for each occurrence was calculated with the Georeferencing Calculator (Wieczorek and Wieczorek 2015).

## Geographic coverage

### Description

**Figure 1.**
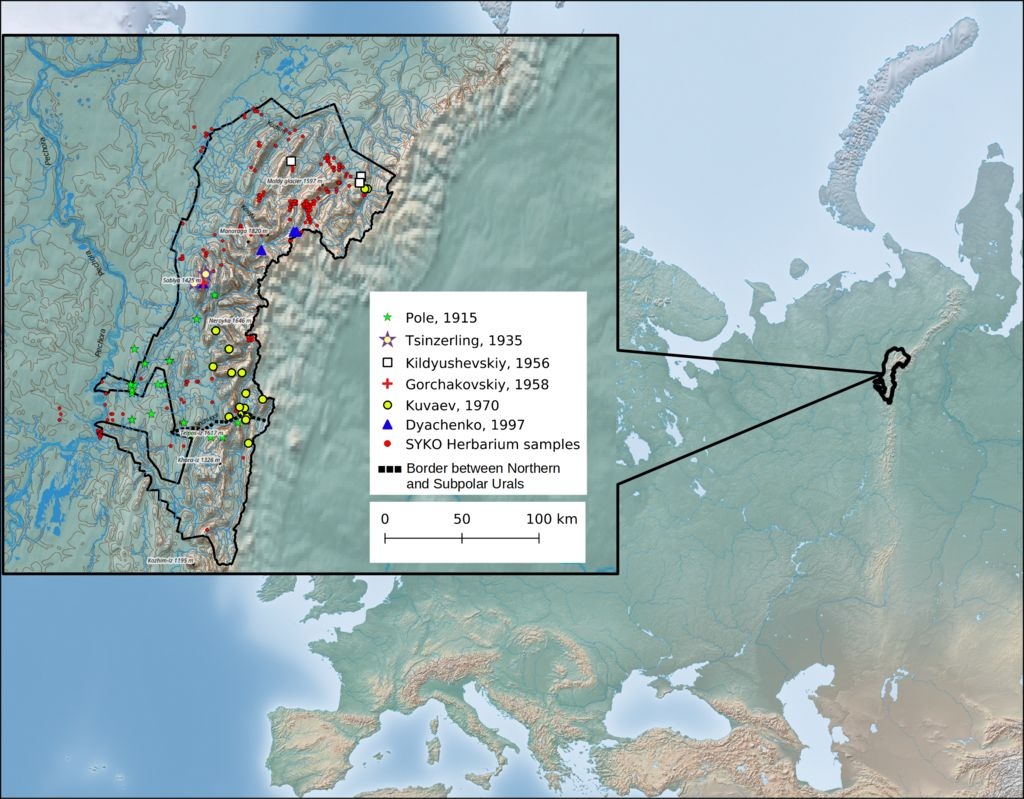
The location of Yugyd Va National Park and moss sampling localities within the park. The collection locations of the preserved samples from the Herbarium (SYKO) examined are shown as red dots, while the localities of data obtained from literature are shown as other symbol types. The base map was made with Natural Earth (naturalearthdata.com). The contour relief and river data for Yugyd Va National Park region were obtained from the Institute of Biology of Komi Scientific Centre of the Ural Branch of the Russian Academy of Sciences (ib.komisc.ru). The map was prepared for publication by the authors of the article.

The length of Yugyd Va National Park is 280 km from North to South and 120 km from West to East. It is located on the western macroslopes of the Subpolar and Northern Urals (Fig. 1).

The Subpolar Urals are part of the Ural Mountains and run from the headwaters of the Lyapin (Khulga) River in the north (65°40'N) to Telposiz Mountain in the south (64°0'N). The mountainous area covers about 32,000 km^2^. There are two main watersheds of the Subpolar Urals: Narodo-Itinsky in the east, with a length of more than 100 km and Issledovatelsky in the west, with a length of more than 150 km. The northern continuation of the Issledovatelsky Range is the Rossomakha Ridge. Traces of glaciation are manifested in the large irregularities of these ridges. The slopes of the mountains are composed of stone placers. The Subpolar Urals are characterised by ridges with high altitudes and alpine landforms. The average height of the peaks is 1300-1400 m. The highest point of the Urals is located at Narodnaya Mountain (1,896 m). The Subpolar Urals have a pronounced asymmetry in their slopes: the eastern slopes of the Subpolar Urals gradually pass into the lowland wetlands of the West Siberian Lowland region, whereas the ridges of the western slopes end abruptly at the Pechora Plain (Chibilev 2011, Taskaev 2006).

The Northern Urals begin at the northern foothills of Telposiz Mountain (1,617 m, 63°55'N) and stretch in a southward direction to Lyalinsky Kamen Mountain (851 m, 59°15'N). The Northern Urals are characterised by having a smooth topography with a maximum elevation of not more than 1,619 m above sea level (Telposiz Mountain). Along the western side of the Northern Urals, there is a long strip of foothills, most of which only rise 200-300 m above sea level (Chibilev 2011, Taskaev 2006).

The rivers in these regions mainly flow through a narrow valley in which floodplains are often poorly expressed. The bottoms of the rivers are covered by pebbles and rocks.

The vertical zonation of the Subpolar and Northern Urals consists of four belts: dark coniferous taiga, light forest belt, mountain tundra and a cold goltsy desert belt. The vegetation of the mountainous dark coniferous taiga is formed mainly by *Picea obovata* and *Betula pubescens* mixed with *Abies sibirica* and *Pinus sibirica*. These forests differ from the plain dark coniferous taiga by there being less waterlogging, with a predominance of green moss and herb vegetation types. The vegetation cover of the light forest belt has a high level of diversity. Here, the complex of light forests, bushes and meadows is presented. The upper boundary of forests is formed by *Picea obovata*, *Abies sibirica*, *Pinus sibirica*, *Betula pubescens* and *Larix sibirica*. The timber line in the Southern part of the park passes at an altitude of about 700 m and, in the Northern part, it falls to 400–200 m. Bushes are represented by communities from *Salix* species, *Betula nana, Juniperus sibirica* (in the Southern part of the reserve) and *Duschekia fruticosa* (in the Northern part). Mountain meadows do not cover large areas and establish mainly ecotopes with rich and humid soils at stream runoffs, valleys and the borders of the stony fields. Mountain tundra communities are located at flat plates and mountain terraces of the upper part of slopes. The cold goltsy desert belt in the Subpolar Urals begins at 300–700 m, in the Northern Urals – at 1000–1100 m (Chibilev 2011, Taskaev 2006).

The climate in this part of the Urals is strongly continental. The meridional location of the ridges has a significant effect on the climate by preventing the movement of moist air masses from west to east. The Subpolar and Northern Urals are distinguished from other parts of the Urals by the relatively abundant precipitation they receive (up to 1,500 mm per year). Most of the precipitation falls in the summer months, from June to August (40-50%). In winter, about 30-40% of the annual precipitation falls. The average monthly temperature of the coldest month (January) in these mountains ranges from -18°C to -20°C, while that of the warmest month (July) is +10°C. The duration of the summer period is 60-75 days (Ponomarev and Pystina 2009)

### Coordinates

63.178 and 65.815 Latitude; 58.712 and 61.479 Longitude.

## Taxonomic coverage

### Description

All mosses were identified to species.

**Figure 2.**
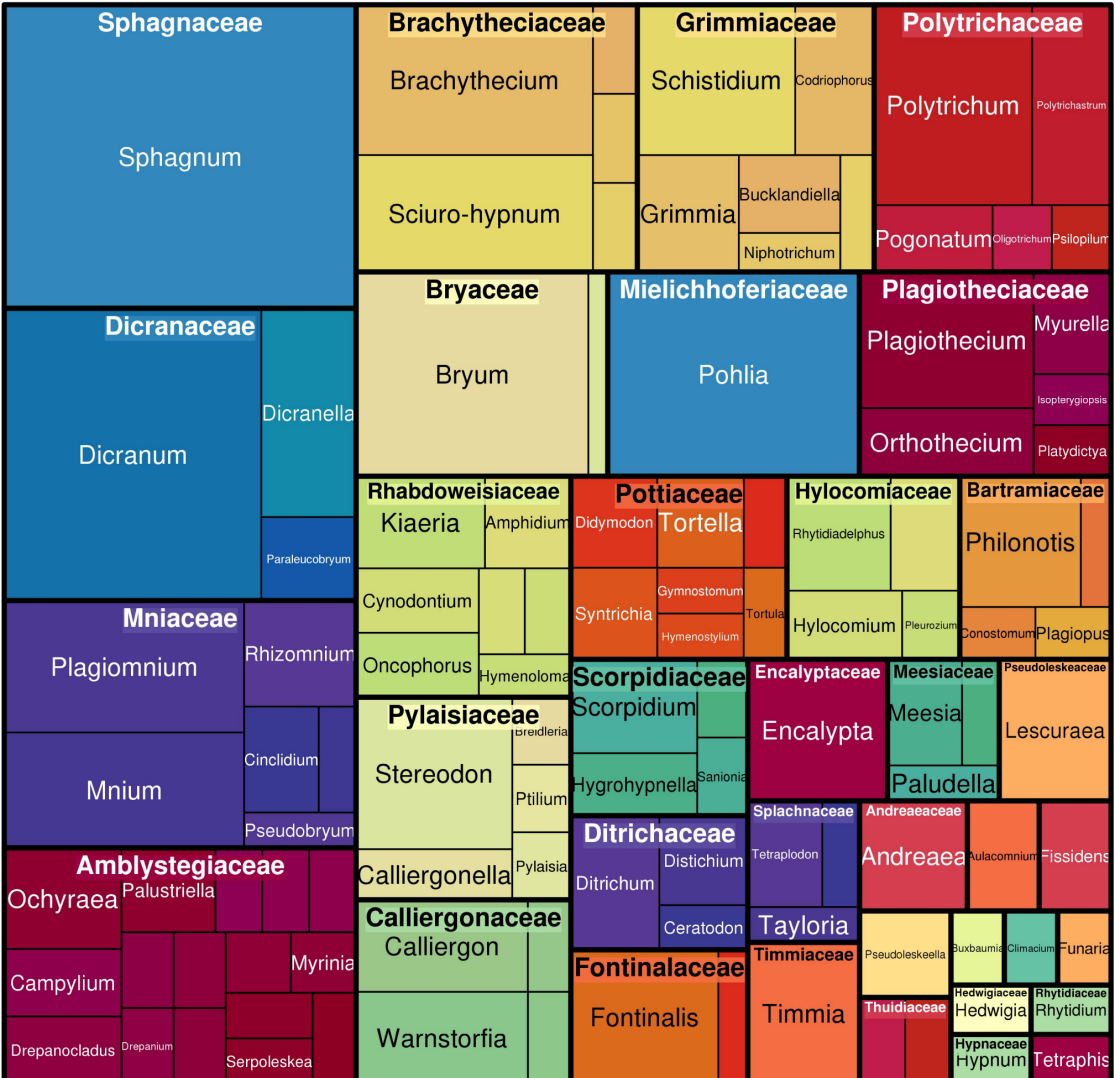
Taxonomic distribution of species amongst moss families in the dataset. The figure was prepared with the “treemap” package in R (Tennekes 2017)

**Figure 3.**
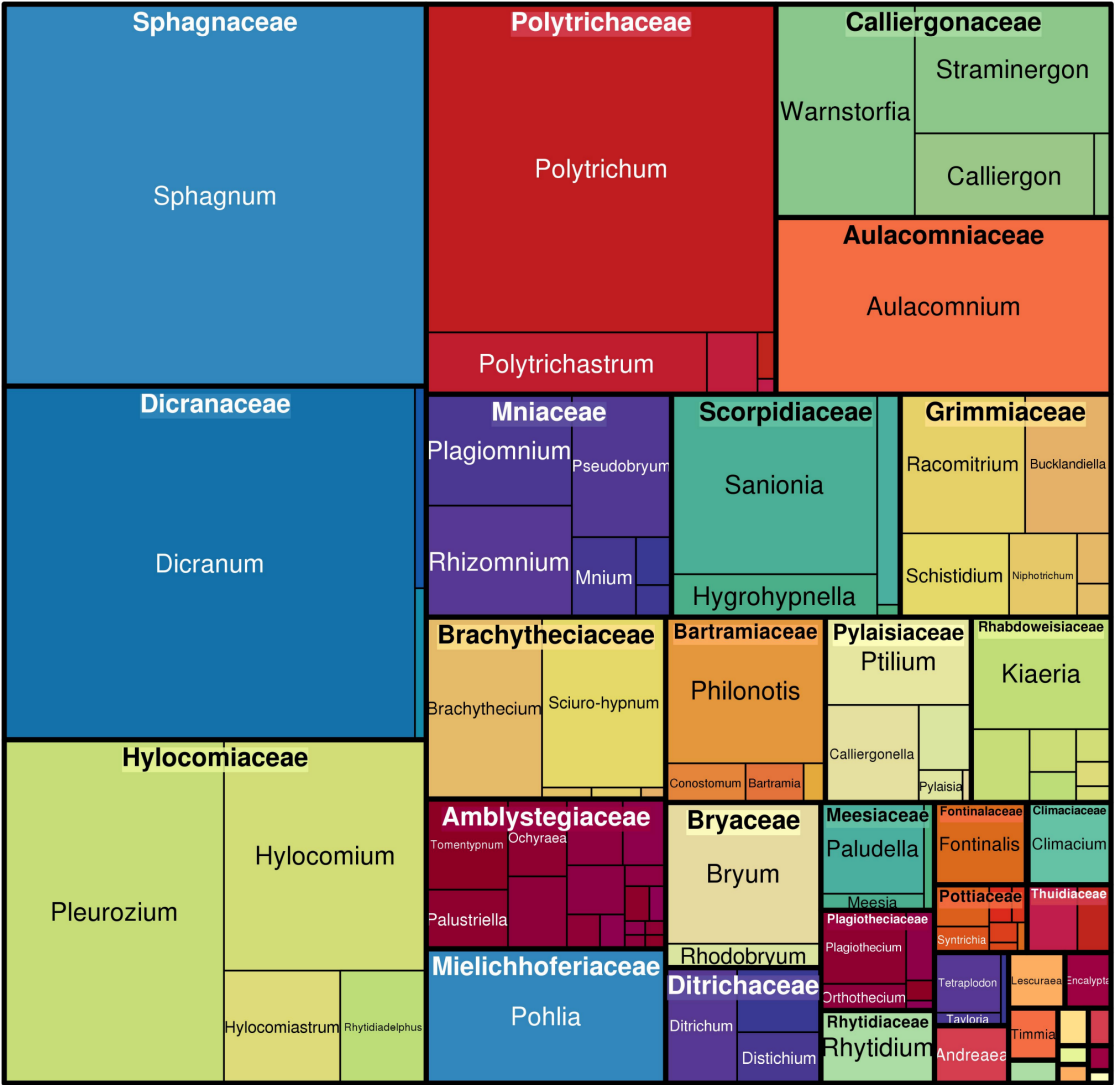
Taxonomic distribution of occurrences amongst moss families in the dataset. The figure was prepared with the “treemap” package in R (Tennekes 2017).

The coverage of the checklist spans the phylum Bryophyta. The highest number of records are from the Bryopsida (84.5%), followed by the Sphagnopsida (8.9%), Polytrichopsida (5.3%), Andreaeopsida (1%) and Tetraphidopsida (0.3%). The top ten families, in terms of the number of species included, comprise about 62% of the species. The top ten families, in terms of the number of occurrences recorded, comprise 77% of the occurrences (Table 2). Of these two groups of top ten families, 60% were included in both groups (Figs 2, 3).

The moss flora of Yugyd Va National Park contains a high number of species, especially taking into account the fact that surveys of the whole territory have not yet been concluded because of the inaccessibility of the region. Although the present survey cannot be considered as complete, the high number of recorded taxa, 302 species belonging to 112 genera and 36 families, already points towards richness of the moss flora of the area. Most of the species are typical members of the Boreal and Holarctic bryoflora and endemic taxa were not recorded. According to available data, the known moss flora of Yugyd Va National Park comprises 60% of the entire known moss flora of the Komi Republic (Zheleznova and Shubina 2012), 25.6% of that of Russia (Ivanov et al. 2017), 16.3% of that of Europe and 1.5% of that of the world (GBIF.org 2018). In the investigated territory, 17 rare species of mosses that are included in the Red Data Book of the Komi Republic (Taskaev 2009) have been revealed and three of these species (*Stereodon plicatulus*, *Bryum rutilans* and *Pseudocalliergon lycopodioides*) are included in the Red Data Book of European Bryophytes (European Committee for the Conservation of Bryophytes (ECCB) 1995).

### Taxa included

**Table:** 

Rank	Scientific Name	
phylum	Bryophyta	
class	Bryopsida	
class	Sphagnopsida	
class	Polytrichopsida	
class	Tetraphidopsida	
class	Andreaeopsida	
family	Amblystegiaceae	
family	Andreaeaceae	
family	Aulacomniaceae	
family	Bartramiaceae	
family	Brachytheciaceae	
family	Bryaceae	
family	Buxbaumiaceae	
family	Calliergonaceae	
family	Climaciaceae	
family	Dicranaceae	
family	Ditrichaceae	
family	Encalyptaceae	
family	Fissidentaceae	
family	Fontinalaceae	
family	Funariaceae	
family	Grimmiaceae	
family	Hedwigiaceae	
family	Hylocomiaceae	
family	Hypnaceae	
family	Meesiaceae	
family	Mniaceae	
family	Mielichhoferiaceae	
family	Plagiotheciaceae	
family	Polytrichaceae	
family	Pottiaceae	
family	Pseudoleskeaceae	
family	Pylaisiaceae	
family	Pseudoleskeellaceae	
family	Rhabdoweisiaceae	
family	Rhytidiaceae	
family	Scorpidiaceae	
family	Sphagnaceae	
family	Splachnaceae	
family	Tetraphidaceae	
family	Thuidiaceae	
family	Timmiaceae	

## Traits coverage

### Data coverage of traits

PLEASE FILL IN TRAIT INFORMATION HERE

## Temporal coverage

### Notes

1905-07-01 through 2015-07-30

## Collection data

### Collection name

Научный гербарий Института биологии Коми НЦ УрО РАН (SYKO). Коллекция мохообразных/Scientific Herbarium of the Institute of Biology Komi Science Centre

### Parent collection identifier

http://ckp-rf.ru/usu/507466/?sphrase_id=7852290

### Specimen preservation method

dried аnd pressed

## Usage rights

### Use license

Other

### IP rights notes

This work is licensed under a Creative Commons Attribution (CC-BY) 4.0 License.

## Data resources

### Data package title

Moss occurrences in Yugyd Va National Park, Subpolar and Northern Urals, European North-East Russia

### Resource link

http://ib.komisc.ru:8088/ipt/resource?r=mosses_occurrence_yugyd_va

### Alternative identifiers

doi.org/10.15468/kfeugm

### Number of data sets

1

### Data set 1.

#### Data set name

Moss occurrences in Yugyd Va National Park, Subpolar and Northern Urals, European North-East Russia

#### Data format

Darwin Core

#### Number of columns

30

#### Character set

utf8

#### Download URL

http://ib.komisc.ru:8088/ipt/resource?r=mosses_occurrence_yugyd_va&v=1.4

#### Description

This study produced a dataset containing information on moss occurrences in the territory of Yugyd Va National Park, located in the Subpolar and Northern Urals, European North-East Russia. The dataset summarises occurrences noted by long-term bryological explorations in remote areas of the Subpolar and Northern Urals from 1943 to 2015 and from studies published since 1915. The dataset consists of 4,120 occurrence records. The occurrence data were extracted from herbarium specimen labels (3,833 records) and data from published literature (287 records). Most of the records (4,104) are georeferenced.

**Data set 1.** 

Column label	Column description
occurrenceID	An identifier for the Occurrence (as opposed to a particular digital record of the occurrence).
institutionID	An identifier for the institution having custody of the object(s) or information referred to in the record. The identifier was taken from the Global Registry of Biodiversity Repositories (http://grbio.org).
collectionCode	The name, acronym, coden or initialism identifying the collection or dataset from which the record was derived. The identifier was taken from registry such as the Global Registry of Biodiversity Repositories
cаtаlogNumber	An identifier (preferably unique) for the record within the dataset or collection.
associatedReferences	A list (concatenated and separated) of identifiers (publication, bibliographic reference, global unique identifier, URI) of literature associated with the Occurrence.
basisOfRecord	Recommended best practice is to use the standard label of one of the Darwin Core classes.
kingdom	The full scientific name of the kingdom in which the taxon is classified.
phylum	The full scientific name of the phylum or division in which the taxon is classified.
class	The full scientific name of the class in which the taxon is classified.
family	The full scientific name of the family in which the taxon is classified.
genus	The full scientific name of the genus in which the taxon is classified.
scientificName	The full scientific name, with authorship and date information, if known. When forming part of an Identification, this should be the name in the lowest level taxonomic rank that can be determined. This term should not contain identification qualifications, which should instead be supplied in the IdentificationQualifier term.
specificEpithet	The name of the first or species epithet of the scientificName.
scientificNameAuthorship	The authorship information for the scientificName formatted according to the conventions of the applicable nomenclaturalCode.
infraspecificEpithet	The name of the lowest or terminal infraspecific epithet of the scientificName, excluding any rank designation.
taxonRank	The taxonomic rank of the most specific name in the scientificName.
country	The name of the country or major administrative unit in which the Location occurs.
countryCode	The standard code for the country in which the Location occurs.
recordedBy	A person, group or organisation responsible for recording the original Occurrence.
day	The integer day of the month on which the Event occurred.
month	The ordinal month in which the Event occurred.
year	The four-digit year in which the Event occurred, according to the Common Era Calendar.
locality	The specific description of the place. Less specific geographic information can be provided in other geographic terms (higherGeography, continent, country, stateProvince, county, municipality, waterBody, island, islandGroup). This term may contain information modified from the original to correct perceived errors or standardise the description.
identifiedBy	A list (concatenated and separated) of names of people, groups or organisations who assigned the Taxon to the subject.
decimalLatitude	The geographic latitude (in decimal degrees, using the spatial reference system given in geodeticDatum) of the geographic centre of a Location. Positive values are north of the Equator, negative values are south of it. Legal values lie between -90 and 90, inclusive.
decimalLongitude	The geographic longitude (in decimal degrees, using the spatial reference system given in geodeticDatum) of the geographic centre of a Location. Positive values are east of the Greenwich Meridian, negative values are west of it. Legal values lie between -180 and 180, inclusive.
coordinatePrecision	A decimal representation of the precision of the coordinates given in the decimalLatitude and decimalLongitude.
coordinateUncertaintyInMetres	The horizontal distance (in metres) from the given decimalLatitude and decimalLongitude describing the smallest circle containing the whole of the Location. Leave the value empty if the uncertainty is unknown, cannot be estimated or is not applicable (because there are no coordinates). Zero is not a valid value for this term.
georeferencedBy	A list (concatenated and separated) of names of people, groups or organisations who determined the georeference (spatial representation) for the Location.
geodeticDatum	The ellipsoid, geodetic datum or spatial reference system (SRS) upon which the geographic coordinates given in decimalLatitude and decimalLongitude is based.

## Additional information

Zheleznova G, Shubina T, Degteva S, Rubtsov M, Chadin I (2018) Moss occurrences in Yugyd Va National Park, Subpolar and Northern Urals, European North-East Russia. v1.3. Institute of Biology of Komi Scientific Centre of the Ural Branch of the Russian Academy of Sciences. Dataset/Occurrence. http://ib.komisc.ru:8088/ipt/resource?r=mosses_occurrence_yugyd_va&v=1.3

## Supplementary Material

Supplementary material 1 Mosses checklist of Yugyd Va National Park, Subpolar and Northern Urals, European North-East Russia Data type: species checklist
Brief description: This file contain checklist of moss flora of Yugyd Va National Park, located in the Subpolar and Northern Urals, European North-East Russia (Russian Federation, Komi Republic). It summarises data noted by long-term bryological explorations in remote areas of the Subpolar and Northern Urals from 1943 to 2015 and from studies published since 1915.
File: oo_275862.xls Zheleznova G, Shubina T, Chadin I

## Figures and Tables

**Table 1.** Data sources for the dataset of moss occurrences in Yugyd Va National Park.

**Data source**	**Number of records**	**Number of species**
Dyachenko 1997	8	5
Dyachenko and Fomicheva 1986	1	1
Gorchakovskiy 1958	2	2
Kildyushevskiy 1956	37	36
Kuvaev 1970	65	48
Pole 1915	171	98
Tsinzerling 1935	3	3
Number of species not present in the Herbarium (SYKO) collection		37
**Subtotal**	**287**	**146**
Herbarium collection	3833	265
**Total**	**4120**	**302**

**Table 2.** Taxonomic distribution of species and species occurrences amongst families in the dataset. Families are listed in order of the total number of their species included in the dataset. Taxonomy follows Ignatov et al. 2006.

**Family**	**Number of species**	**Records**
Sphagnaceae	27	560
Dicranaceae	26	512
Mniaceae	22	190
Amblystegiaceae	21	126
Brachytheciaceae	19	149
Grimmiaceae	16	162
Polytrichaceae	16	475
Bryaceae	13	88
Mielichhoferiaceae	13	107
Plagiotheciaceae	13	40
Calliergonaceae	10	248
Hylocomiaceae	7	502
Scorpidiaceae	7	175
Aulacomniaceae	2	207
**Subtotal**	**212**	**3541**
**Total (including other 22 families)**	**302**	**4120**
